# Active Trachoma among Children in Mali: Clustering and Environmental Risk Factors

**DOI:** 10.1371/journal.pntd.0000583

**Published:** 2010-01-19

**Authors:** Mathieu Hägi, Jean-François Schémann, Frédéric Mauny, Germain Momo, Doulaye Sacko, Lamine Traoré, Denis Malvy, Jean-François Viel

**Affiliations:** 1 CNRS UMR 6249 “Chrono-Environment”, Faculty of Medicine, Besançon, France; 2 University of Bordeaux 2 (EA 3677 and Centre René Labusquière), Bordeaux, France; 3 Institute of African Tropical Ophthalmology (IOTA), Bamako, Mali; 4 West African Health Organization, Vision 2020 coordination group, Bobo-Dioulasso, Burkina Faso; Ghana Health Service, Ghana

## Abstract

**Background:**

Active trachoma is not uniformly distributed in endemic areas, and local environmental factors influencing its prevalence are not yet adequately understood. Determining whether clustering is a consistent phenomenon may help predict likely modes of transmission and help to determine the appropriate level at which to target control interventions. The aims of this study were, therefore, to disentangle the relative importance of clustering at different levels and to assess the respective role of individual, socio-demographic, and environmental factors on active trachoma prevalence among children in Mali.

**Methodology/Principal Findings:**

We used anonymous data collected during the Mali national trachoma survey (1996–1997) at different levels of the traditional social structure (14,627 children under 10 years of age, 6,251 caretakers, 2,269 households, 203 villages). Besides field-collected data, environmental variables were retrieved later from various databases at the village level. Bayesian hierarchical logistic models were fit to these prevalence and exposure data. Clustering revealed significant results at four hierarchical levels. The higher proportion of the variation in the occurrence of active trachoma was attributable to the village level (36.7%), followed by household (25.3%), and child (24.7%) levels. Beyond some well-established individual risk factors (age between 3 and 5, dirty face, and flies on the face), we showed that caretaker-level (wiping after body washing), household-level (common ownership of radio, and motorbike), and village-level (presence of a women's association, average monthly maximal temperature and sunshine fraction, average annual mean temperature, presence of rainy days) features were associated with reduced active trachoma prevalence.

**Conclusions/Significance:**

This study clearly indicates the importance of directing control efforts both at children with active trachoma as well as those with close contact, and at communities. The results support facial cleanliness and environmental improvements (the SAFE strategy) as population-health initiatives to combat blinding trachoma.

## Author Summary

Active trachoma is not uniformly distributed in endemic areas, and local environmental factors influencing its prevalence are not yet adequately understood. Determining whether clustering is a consistent phenomenon may help predict likely modes of transmission and help to determine the appropriate level at which to target control interventions. In this work, we estimated the magnitude of clustering at different levels and investigated the influence of socio-economic factors and environmental features on active trachoma prevalence among children in Mali (1996–1997 nationwide survey). Clustering revealed significant results at the child, caretaker, household, and village levels. Moreover, beyond some well-established individual risk factors (age between 3 and 5, dirty face, and flies on the face), we found that temperature, sunshine fraction, and presence of rainy days were negatively associated with active trachoma prevalence. This study clearly indicates the importance of directing control efforts both at children with active trachoma as well as those with close contact, and at communities. These results support facial cleanliness and environmental improvements as population-health initiatives to combat blinding trachoma.

## Introduction

Active trachoma is a chronic, recurrent keratoconjunctivitis caused by ocular infection with the bacterium *Chlamydia trachomatis.* The condition remains hyper-endemic in the most impoverished communities in underdeveloped countries (in parts of Africa, the Middle-East, south and central Asia, Latin America and aboriginal communities in Australia). Beyond the occurrence of scarring and trichiasis sequellae, this disease is considered to be one of the most frequent causes of blindness and loss of vision worldwide, and a leading cause of preventable blindness [1],[2].

Active trachoma is a family-based disease, clustering at the household and community levels [3],[4],[5]. Its distribution also shows strong spatial patterns. For example, data from baseline survey found trachoma prevalence ranges from 9 to 45% in Burkina-Faso [6], from 23 to 47% in Mali [7], and from 3 to 82% in Niger [8]. No animal reservoir for *C. trachomatis* has been found in endemic environments, the primary reservoir being in children, particularly those under the age of five. The disease is characterized by facile transmission of infected ocular secretions mainly between children and women who care for them. Transmission is thought to occur via fingers, fomites (such as shared cloths, towels or bed sheets), coughing and sneezing, and eye-seeking flies (mainly *Musca sorbens*, living in close proximity to humans and acting as passive vectors, frequently feeding on ocular and nasal discharges). However, the relative importance of these routes has not been ascertained [9].

Since 1997, trachoma has been a priority disease targeted by the World Health Organization (WHO), with the establishment of the Alliance for Global Elimination of Trachoma; the Alliance's goal is to eliminate blinding trachoma before the year 2020, using the so-called ‘SAFE’ strategy [10]. This strategy, based on the current understanding of trachoma epidemiology, includes surgery of trichiasis sequellae (S), antibiotic treatment of active disease (A), face washing and personal hygiene improvement (F), and environmental changes to reduce transmission (E).

Although encompassed in the SAFE strategy, local environmental factors influencing active trachoma prevalence are not yet adequately understood. Water supply, fecal and refuse disposal, presence of animal pens within human households, and fly density are all potentially modifiable risk factors for the disease [11]. Other environmental factors could also indirectly influence trachoma transmission. The abundance of *M. sorbens* (and its relationship to active trachoma prevalence) seems indeed to vary in time and space, and it could depend on meteorological conditions, the presence or absence of breeding sites, or the nature and extension of vegetation cover.

Understanding the role of heterogeneity in the environment and in behavior remains a major challenge [12]. Determining whether clustering is a consistent phenomenon may help predict likely modes of transmission, and determine the appropriate level at which to target control interventions. Clustering implies that observations within a particular cluster are more alike than observations from different clusters, due to the shared social and geographical environment experienced within the cluster. In a similar way, spatial correlation occurs when individuals living in close proximity are more likely to have the same infectious status than individuals living farther apart. Left unaddressed, both effects cause standard errors to be underestimated, leading to incorrect inferences.

The aims of this study were therefore to disentangle the relative importance of clustering at different levels and to assess the respective role of individual, socio-demographic, and environmental factors on active trachoma prevalence among children in Mali, while accounting for clustering effects and spatial correlation.

## Materials and Methods

### Study population

We used anonymous data from the Mali national trachoma survey carried out in 1996–1997. The framework of this survey has been fully described elsewhere [7],[13]. Briefly, the sampling method was based on a two-level, cross-sectional random cluster sample design, using procedures suggested by the WHO program for prevention of blindness [14]. Mali is divided into seven administrative regions (excluding the capital Bamako). The sampling frame was the list of villages drawn up for the 1987 national census, excluding the administrative districts of the capitals of the regions. For each of the regions, 30 villages were drawn at random, using the cumulative total method with probability proportional to population size. This method allowed coverage of 210 villages. In each of the selected villages, a sub-sample of households was drawn at random. Every child under 10 years of age in the randomly chosen households was examined. Active trachoma was graded as follicular or intense according to the WHO simplified grading system [15]. The five ophthalmologists who examined were trained by an expert and their accuracy at applying the diagnostic scheme was verified before beginning the survey (κ statistic ≥0.80).

### Risk factors

Malian traditional social structure exhibits levels of clustering. Children are nested within caretakers (usually the biological mother, but also a sister, or the mother of other children), who are nested within households (defined as people sharing a common doorway), that are nested within villages. During the 1996–1997 national prevalence survey, data were collected at the different levels of this hierarchical structure.

For each child, presence of active trachoma in either eye, facial cleanliness, and presence of flies on child's face were assessed during clinical examination. Information about age, and sex was also collected. Each mother or child's caretaker was questioned about her educational level, the number of children she took care of, the hygiene habits (frequency of body washings, quantity of water used, use of soap, wiping after washing, number of times the child had his/her face washed), and the distance from usual water source. Further information was collected at the household level: number of children, number of people sleeping in the same room, building and roof material, common ownership of goods or animals (radios, bikes, motorbikes, carts, ploughs, traction bulls, donkeys, cattle and small ruminants), presence of a well, latrines, or animal stables, and educational level of the household's head. At the village level, demographic (population size, ethnic groups), structural (water sources, distance to a medical center, distance to a health post, presence of a pharmacy), social (presence of a school, existence of a women's association), and economic (water sources, primary agricultural products) information was elicited.

Ethical clearance for the initial study (1996–1997) was granted by the Institute of African Tropical Ophthalmology (Bamako, Mali) ethical committee. All data collection activities were carefully explained to, and oral consent was obtained from traditional authorities and heads of households. Written consent was not obtained and oral consent was not specifically documented because the survey was considered by the ethical committee as part of the monitoring and evaluation of routine health activities carried out by the Malian Ministry of Health. According to French law, the present observational study, consisting of the reanalysis of anonymous data, does not need the approval of an Institutional Review Board/Independent Ethics Committee [16].

In addition to data collected during the field survey, environmental variables were retrieved later from various databases at the village level. First, villages were geo-referenced using the GEOnet Names Server that provides access to foreign geographic feature names (with WGS 84 coordinate system) (GEOnet Names Server National Geospatial-Intelligence Agency, Bethesda, MD, USA). Average monthly climatic conditions at village locations (corresponding to the month of field-data collection for a given village) were estimated for the 1961–2000 time period. In an exploratory approach, we used data from the Food and Agricultural Organization (FAO) agroclimatic database (FAOCLIM), and the New_LocClim software (with the Shepard interpolation method) to calculate the following indicators: continentality index, Koeppen class, Budyko index, rainfalls, sunshine fraction, temperature (mean, minimum and maximum per day), water vapor pressure, wind speed, number of rainy days, and number of months without any rainfall (ftp://ext-ftp.fao.org/SD/Reserved/Agromet/New_LocClim/). Annual means were also calculated for the same meteorological indicators. Villages' altitude was also available from this database with a 10km precision. Normalized Difference Vegetation Index (NDVI) data have been obtained from the Vegetation Program (http://free.vgt.vito.be), using data from the French Space Agency (CNES). Annual mean and maximum NDVIs were calculated and extracted from a circular buffer of 25 km^2^ around each village. Irrigated farming system, as a proxy for hydrology system, has been established from a map of African agricultural systems published by the FAO (http://www.fao.org/farmingsystems/). All geo-referenced data were constructed and analyzed using ArcView 8 GIS (ESRI, Redlands, CA, USA).

### Statistical analysis

The outcome (active trachoma) was coded as a binary variable (presence/absence). Continuous independent variables were modeled as categorical variables (tertiles). Variables with missing data exceeding a proportion of 10% were excluded from the analyses. For the remaining variables, an extra “missing value” category was defined. The data for analysis form a hierarchical structure with four levels: child (ijkl), caretaker (ijk), household (ij), and village (i). Multilevel random logistic regression models were carried out with the following strategy.

To demonstrate clustering of active trachoma at different levels, we developed a “null” random effect model in the Bayesian framework (to allow for a potential spatial correlation):
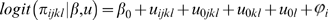
(1)


*β_0_*: fixed effect intercept,

*u_ijkl_*: child random effect,

*u_0jkl_*: caretaker random effect,

*u_0kl_*: household random effect,

*u_0l_*: village random effect (or uncorrelated heterogeneity),


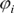
: spatial clustering or correlated heterogeneity.

For the spatial component, a conditional autoregressive (CAR) structure was used.
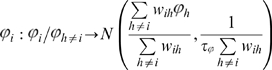
(2)


Following Boyd et al. [17], we considered distance-based weights (w_ih_) for the CAR structure. Since each sampling unit must have at least one neighbor, villages within 128 km of a given village - the minimum distance required to provide all villages with at least one neighbor - were considered neighbors. Dropping each random effect in turn, we kept the model that showed the best goodness of fit (i.e., the lowest deviance). Then we calculated the 95% credibility interval of the individual baseline probability of infection according to the formulae:
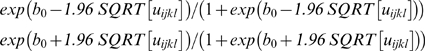
(3)


To investigate risk factors for active trachoma, we first introduced each field-collected variable in turn into the best “null” model (univariate analyses). In a second step, a forward selection was applied to the subset of field-collected variables that had a *p* value of 0.20 or less in the univariate analyses. This forward stepwise procedure was run within each level, starting from the lowest to the highest level of the best “null” model. Because Bayesian software are slower for models that can also be run in MLwiN (the latter being also more user-friendly), the initial covariate selection process was conducted using MLwiN [18]. We used the Iterative Generalized Least Square (IGLS) algorithm, relaxing the binomial assumption by including extra binomial variation. In a third step, this initial hierarchical multivariate model was enriched by considering the environmental variables (all assigned to villages) in a further stepwise procedure, yielding a final multilevel IGLS model:
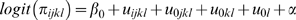
(4)with: *β_0_*: fixed effect intercept,

*u_ijkl_ = σ_o_^2^ π_ijkl_ (1 - π_ijkl_)*: child random effect,

*σ_o_^2^*: extra-binomial parameter,

*π_ijkl_*: probability of observing an active trachoma,

*u_0jkl_*: caretaker random effect,

*u_0kl_*: household random effect,

*u_0l_*: village random effect,

*α = Σ βx_ijkl_ + Σ βx_jkl_ + Σ βx_kl_ + Σ βx_l_*

*βx_ijkl_*: child fixed effects,

*βx_jkl_*: caretaker fixed effects,

*βx_kl_*: household fixed effects,

*βx_l_*: village fixed effects.

Finally, an equivalent Bayesian model was created by incorporating prior distributions for each of the unknown parameters in the model, and performing inference on the resulting posterior distributions. Its form was:
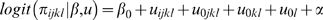
(5)


Bayesian models were run with WinBUGS software [19]. Because we wanted the observations, not the priors, to drive the conclusions, we chose weak priors:

- uniform priors for fixed parameters,
- gaussian distribution for the random effects: u|σ^2^_u_∼N(0, σ^2^_u_),
- gamma distribution for hyperparameters: σ^2^_u_∼Γ(0.001, 0.001),
- gamma distribution for CAR hyperparameter: τ_φ_∼Γ(0.001, 0.001).

Two parallel chains were run. A burn-in of 1,000 iterations was allowed, followed by 10,000 iterations for which parameter values were stored. Bayesian summary statistics, density plots, and plots for convergence diagnosis were produced with the CODA (COnvergence Diagnosis and output Analysis) package (http://cran.r-project.org/web/packages/coda/index.html). The diagnosis (based on chain history, posterior density plots, and plots of Gelman & Rubin tests) provided no reason to suspect lack of convergence.

Odds ratios (ORs) and their 95% confidence intervals (CIs) in the frequentist framework, or their credibility intervals (CIs) in the Bayesian framework were then calculated.

## Results

**Figure 1:**
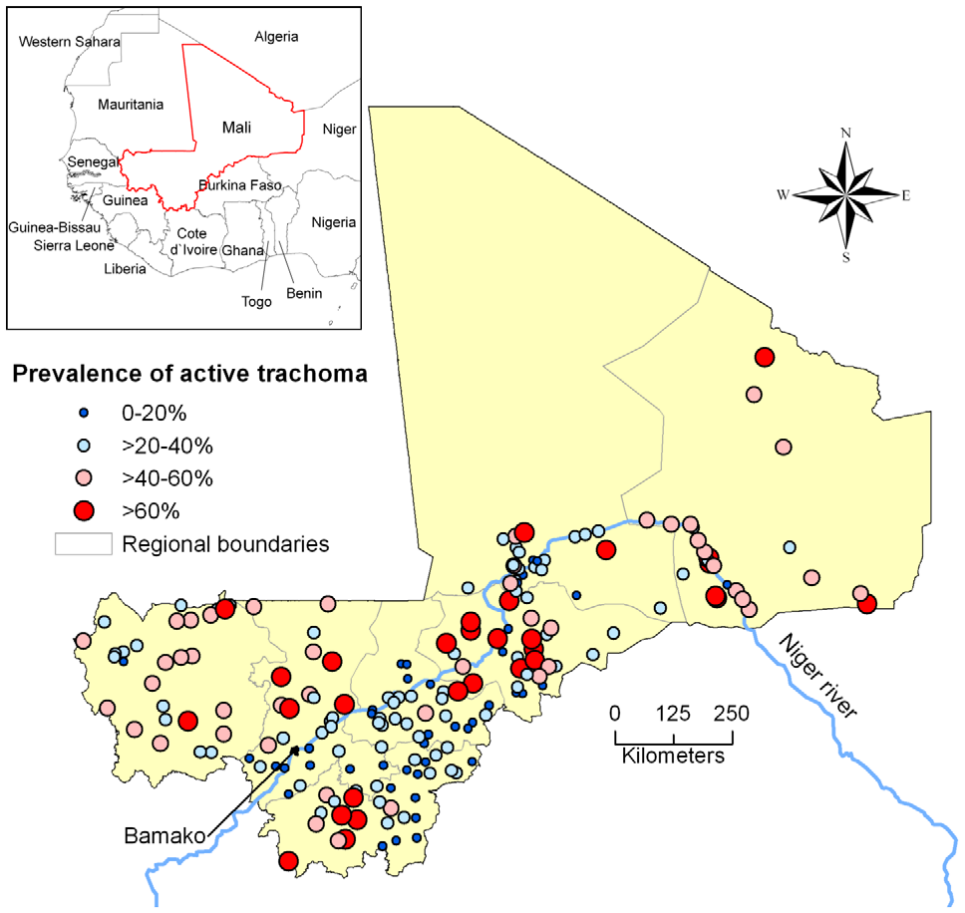
Prevalence of active trachoma among children in Mali (203 villages, 1996–1997).

Two hundred and three villages were unambiguously traced and precisely geo-referenced in the “GEOnet Names Server” database, corresponding to 2,269 households, 6,251 caretakers, and 14,627 children under the age of 10. Overall, 5,273 children had active trachoma during the 1996–1997 baseline survey, yielding a national estimate of active trachoma (weighted according to regional populations) of 35.0% (95% CI 34.2%–35.8%). Figure 1 displays the prevalence of active trachoma among children per village (ranging from 0.0% to 88.1%).

The best “null” model included the four level-specific random effects but excluded the spatial correlation (*p* value = 0.26 in the five random effect model), meaning that the risk of active trachoma in any village did not depend on neighboring villages. Table 1 shows the random effects variances estimated from this four-level “null” model, and their recalibration to 100 percent. All variance components were highly statistically significant. The higher proportion (36.7%) of the variation in the occurrence of active trachoma was attributable to village-level differences. A substantial portion of the total variation in response also occurred between children (within caretakers, 24.7%), and between caretakers (within households, 25.3%). With an intercept of −0.919, 95% of recruited children had an individual baseline probability of active trachoma that ranged from 0.05 to 0.75, highlighting substantial heterogeneity in the individual baseline risk of infection.

**Table 1:** Random effects variances of active trachoma among children from the best multilevel “null” model (Mali, 1996–1997).

*Level*	σ^2^ *	SD†	*p- value*	%
*Child*	1.052	0.311	<10^−3^	24.7
Caretaker	1.081	0.166	<10^−10^	25.3
Household	0.566	0.096	<10^−8^	13.3
Village	1.564	0.233	<10^−10^	36.7

*: Random effect variance.

**†:** Standard deviation.

Twenty-five variables were dismissed because of missing data exceeding the proportion of 10%. Forty-eight field-collected and 28 environmental variables were therefore analyzed in univariate models. All these variables can be found as supporting information, available at PloS Neglected Tropical Diseases online (Table S1). Many field-collected variables were linked with active trachoma prevalence (*p* value≤0.20), as well as latitude, longitude, NDVI and the vast majority of climatic variables.

The final model consisted of seven field-collected and four environmental variables (Table 2). In the Bayesian framework, three individual fixed effects were statistically significant: age between 3 and 5 (OR = 2.17, 95% CI 1.88–2.49), dirty face (OR = 4.91, 95% CI 3.99–6.03), and flies on the face (OR = 2.85, 95% CI 2.18–3.73). At the caretaker level, a reduced risk was associated with wiping after washing (OR = 0.72, 95% CI 0.59–0.88). Household with radio (OR = 0.77, 95% CI 0.63–0.94), and household with motorbike (OR = 0.86, 95% CI 0.71–1.04) were negatively associated with active trachoma prevalence, although not significantly so for the latter. Finally, one village-level field-collected variable was statistically significant: presence of a women's association in the village (OR = 0.55, 95% CI 0.36–0.84). Average monthly maximal temperature and sunshine fraction, and average annual mean temperature were negatively associated with active trachoma prevalence (with the exception of the third tertile of mean maximal temperature) (Table 2). The presence of rainy days was of borderline significance (OR = 0.57, 95% CI 0.31–1.07). No “missing values” category was statistically significant (results not shown). All level-specific random effects were statistically significant (Table 3). Not surprisingly, variance occurring at all levels (but the caretaker level) decreased when introducing the corresponding risk factors. In the frequentist framework (IGLS), point estimates were slightly lower at the child level, but similar at the other levels (although the number of rainy days became statistically significant).

**Table 2:** Active trachoma prevalence odds ratios (95% confidence interval for IGLS* model, 95% credibility interval for Bayesian models) in multilevel models including field-collected and environmental variables (fixed effects).

Variables		Category	IGLS	BHM†
**Field-collected variables**	**Child level**			
	Age (years)	<3	1	1
		3 to 5	1.91 (1.75–2.08)	2.17 (1.88–2.49)
		6 to 10	0.98 (0.89–1.08)	1.00 (0.87–1.14)
	Dirty face	No	1	1
		Yes	3.73 (3.37–4.13)	4.91 (3.99–6.03)
	Flies on the face	No	1	1
		Yes	2.36 (1.95–2.85)	2.85 (2.18–3.73)
	**Caretaker level**			
	Wiping after washing	No	1	1
		Yes	0.76 (0.65–0.89)	0.72 (0.59–0.88)
	**Household level**			
	Radio	No	1	1
		Yes	0.81 (0.69–0.95)	0.77 (0.63–0.94)
	Motorcycle	No	1	1
		Yes	0.89 (0.75–1.04)	0.86 (0.71–1.04)
	**Village level**			
	Women's association	No	1	1
		Yes	0.60 (0.45–0.81)	0.55 (0.36–0.84)
*Environmental variables*	*Average monthly conditions*			
	Maximum temperature	<34.6°C	1	1
		34.6–38.7°C	0.56 (0.37–0.87)	0.51 (0.29–0.90)
		>38.7°C	1.02 (0.61–1.70)	1.03 (0.51–2.05)
	Sunshine fraction	<62.8%	1	1
		62.8–69.9%	0.66 (0.47–0.92)	0.61 (0.41–0.91)
		>69.9%	0.55 (0.38–0.79)	0.50 (0.32–0.79)
	Number of rainy days	0	1	1
		1 and more	0.63 (0.41–0.97)	0.57 (0.31–1.07)
	**Average annual conditions**			
	Mean temperature	<27.3°C	1	1
		27.3–28.1°C	0.54 (0.40–0.74)	0.49 (0.32–0.74)
		>28.1°C	0.63 (0.45–0.89)	0.57 (0.36–0.90)

*: Iterative generalized least square.

**†:** Bayesian hierarchical model.

**Table 3:** Random effects variances of active trachoma among children from the multivariate Bayesian model (Mali, 1996–1997).

Level	σ^2^ *	SD†	*p- value*	%
Child	0.652	0.340	0.06	20.8
Caretaker	1.112	0.193	<10^−8^	35.4
Household	0.456	0.086	<10^−6^	14.5
Village	0.920	0.157	<10^−8^	29.3

*: Random effect variance.

**†:** Standard deviation.

## Discussion

This study confirms the role of individual and socio-demographic factors on active trachoma prevalence. It goes further, however, by identifying clustering (at four different hierarchical levels), and by highlighting four environmental risk factors, using sound statistical techniques.

The 1996–1997 national survey was large, including about 15,000 children, and benefited from a very high participation rate, minimizing selection bias. The simplified WHO grading system is a well-established tool for outcome definition, and examinations were conducted by well-trained ophthalmologists. This dataset has already been analyzed at the individual level with traditional statistical tools [13],[20]. However, some prevalence patterns seemed strongly dependent on local spatial factors possibly related to specific spatial behavioral factors. Hierarchical modeling was therefore planned to simultaneously analyze the influence of individual and environmental factors [20].

One limitation of this study is that sampling weights were not incorporated in the multilevel regression models. There is currently no well established general multilevel consistent estimation method, the proposed methods applying only to certain multilevel models and parameters [21]. Researchers have, therefore, still to make a choice between estimating a multilevel model or estimating a single level model but incorporating the sampling design in the standard error estimation. However, since we have adjusted for cluster indicators at the different levels, we assumed near-zero correlations between active trachoma occurrence and the selection probabilities (depending on the household size or the number of households per village), minimizing any potential bias [22].

Our results should be interpreted with the following points in mind. First, the proportion of individuals with active disease may not correspond to the proportion of individuals with infection, since signs of conjunctival inflammation may concurrently be the result of other bacterial infections or mechanical irritation, and even after infection is eliminated from a community, some individuals may still show signs of active disease [23]. Second, this risk factor study was designed to be simple in order to increase participation and to make it logistically manageable. We therefore did not support the questionnaire with observational data, such as water-use behavior and evidence of latrine use. This may have led to us missing more subtle or complex relationships between risk factors and active trachoma. Third, fixed and random effects were estimated from multilevel analyses of cross-sectional data. Cross-sectional designs, however, provide a weak basis for understanding health determinants and should be complemented with analyses of longitudinal data.

The main contribution of the current work is to disentangle the relative importance of clustering at four different hierarchical levels. This approach compensates for residual variability unaccounted for by fixed effects. Broadly speaking, level-specific random effects reflect a social proximity (while a spatial random effect characterizes a geographical proximity), and provide an estimate of “explanatory” power associated with each level. Clustering of active trachoma disease by household has been shown to occur in a number of communities [4], [5], [24]–[26]. Using a mathematical model of the household transmission of ocular *C. trachomatis*, Blake et al. have recently found that an average of 71% of incident infections were the results of transmission within the household in four endemic populations in The Gambia and Tanzania [23]. On our side, we have demonstrated clustering at the child, caretaker, household, and village levels, which fully explained the spatial patterns in the observations. Child heterogeneity is the result of exposure to unobserved or unknown individual risk factors. The caretaker clustering lends support to intrafamilial transmission, implying spread of disease between individuals with close contact. The aggregation of cases within households (although the magnitude of this effect is small) reflects the social organization at this level, with similar behaviors and socio-economic factors. The village clustering highlights the transmission of trachomatous infection between the members of nearby households. This significant variation from village to village is consistent with the presence of contextual effects, such as social connections, cultural habits, and local environmental factors (possibly influencing the density of domestic flies). The non-significant spatial correlation component means that individuals living in close villages are not more likely to have the same infection status than individuals living further apart, reinforcing the influence of social and environmental factors at the village level.

Regarding fixed effects, the different estimation procedures used by MLwiN and WinBUGS could explain the slightly different results at the lower (child) hierarchical level. Prevalence of active trachoma is known to strongly vary with age [6], [8], [13], [27]–[30], peaking in pre-school children, as shown in our study. Presence of flies has long been associated with risk of active trachoma [31]–[34]. There is good evidence that *M. sorbens* and other domestic muscidae can act as vectors for *C. trachomatis* transmission [3], although insecticide spraying campaigns to control *M. sorbens* have obtained variable results on trachoma prevalence [35],[36]. Poor facial cleanliness has been associated consistently with the disease (see Wright et al. for a review [11]). It is very hard, however, to determine whether a dirty face is a risk factor or a sign of active trachoma. Hygiene practices protect against disease, and more specifically, face washing habits [5], [6], [13], [28], [37]–[41]. In the previous analysis of this dataset [13], strong negative links were found with frequency of body and face washings, and use of soap in a multivariate approach. In our multilevel models, the only significant hygienic practice was wiping the face of children after washing (frequency of body washings was not considered because missing data exceeded a proportion of 10%). This pattern could be explained by the introduction of level-specific random effects, which widens confidence and credibility intervals.

Socioeconomic factors are strongly linked to risk of active trachoma [5]–[7]. In line with these results, we showed that community wealth is negatively linked to trachoma prevalence, as reflected by motorbike or radio ownership (also suggesting access to information and education). The negative association with the presence of a women's association represents the most important programmatic finding. Among strategies to fight active trachoma, personal (e.g., facial cleaning) and environmental hygiene (e.g., water sanitation) are key. A health communication programme directed at improving hygiene behaviors could take advantage of women's associations as communication networks, to promote community awareness and participation in the prevention of active trachoma. Such women's involvement would represent not only a tool for sanitation and disease prevention, but also a tool for empowerment, dignity, self-sufficiency as well as access to education for girls.

Results of the present research indicate that sunshine fraction, land surface temperature, and rainy days independently influence the distribution of active trachoma in Mali, after adjusting for field-collected variables. Environmental factors, and particularly climatic conditions, may be important determinants of fly physiology and behavior [33], [42]–[45]. Sunshine fraction and land temperatures are negatively linked to active trachoma prevalence. Although speculative, the following mechanisms can be envisaged. Preferred breeding sites of *M. sorbens* are fresh human fecal materials lying on the ground. High temperature and sunshine may induce rapid dryness of fecal materials, causing them to become improper breeding sites. Moreover, life duration of *M. sorbens* strongly depends on temperature. Life duration decreases with temperature from 35 days at 24°C to less than 12 days at 32°C, yielding less frequent contacts of *M. sorbens* with children's face, and in turn, lower contamination of *M. sorbens* with *C. trachomatis* [44],[45]. In our study, a high humidity level (as assessed by the number of rainy days) is associated with a lower trachoma prevalence, as already reported by Salim & Scheick [46]. There are a number of plausible explanations for this result, including a lower fly population density (decreasing trachoma transmission), lower conjunctival dryness (disfavoring *Chlamydia* infection), and greater water availability (favoring hygiene practices).

Ideally, control strategies for such communicable diseases should be tailored to the local epidemiology, taking advantage of explained variances that estimate impact. Our results clearly indicate the importance of directing control efforts both at children with active trachoma as well as those with close contact (household level), and at communities (village level). They support facial cleanliness and environmental improvements with education and improved local economy (the parts F and E of the SAFE strategy), as population-health initiatives to combat blinding trachoma.

## Supporting Information

Alternative Language Abstract S1 Translation of the abstract into French by J-FV.
(0.02 MB DOC)

Table S1 Risk factors for active trachoma (children under 10 years, Mali, 1996–1997), according to their hierarchical level and use.
(0.08 MB DOC)
