# Supplementary material for: Active Trachoma among Children in Mali: Clustering and Environmental Risk Factors
Source: PLoS Negl Trop Dis. 2010 Jan 19;4(1):e583. doi: 10.1371/journal.pntd.0000583 (PMC2799671; doi:10.1371/journal.pntd.0000583)
Supplement: Alternative Language Abstract S1 — Translation of the abstract into French by J-FV. (0.02 MB DOC) [file pntd.0000583.s001.doc]

"Trachome actif parmi les enfants du Mali :

agrégation des cas et facteurs de risque environnementaux"

**Etat de la question** : Le trachome actif n'est pas réparti uniformément dans les zones endémiques, et les facteurs environnementaux locaux influençant sa prévalence sont encore imparfaitement compris. La mise en évidence d'une agrégation des cas peut aider à identifier les modes de transmission vraisemblables et à déterminer les niveaux d'intervention les plus appropriés. Les buts de cette étude étaient donc de distinguer l'importance relative du phénomène d'agrégation à différents niveaux d'organisation, et d'évaluer le rôle respectif des facteurs de risque individuels, socio-démographiques et environnementaux sur la prévalence du trachome actif chez les enfants du Mali.

**Méthodes / Principaux résultats** : Nous avons utilisé les données anonymes recueillies à différents niveaux de la structure sociale traditionnelle du Mali, durant l'enquête nationale sur le trachome conduite en 1996-1997 (14627 enfants âgés de moins de 10 ans, 6251 mères, 2269 concessions et 203 villages). En complément des données de terrain, des variables environnementales définies au niveau du village ont été extraites de différentes bases de données. Des modèles de régression logistique bayésiens ont été appliqués à ces données de prévalence et d'exposition. L'agrégation est apparue significative aux quatre niveaux d'organisation étudiés. La variance de la prévalence du trachome actif était principalement expliquée par le niveau "village" (36,7%), puis par les niveaux "concession" (25,3%) et "enfant" (24,7%). Nous avons retrouvé certains facteurs de risque individuels déjà bien établis (âge entre 3 et 5 ans, visage sale, mouches sur le visage). Mais nous avons surtout identifié des facteurs, définis aux niveaux d'organisation supérieurs, significativement associés à une diminution de la prévalence : "mère" (essuyage de l'enfant après de la toilette), "concession" (possession d'un poste radio ou d'une mobylette), et "village" (association de femmes, moyenne mensuelle des températures maximales, moyenne mensuelle de l'ensoleillement, moyenne annuelle des températures, jours de pluie).

**Conclusions / Signification** : Cette étude montre clairement l'importance de diriger les efforts de contrôle, tant vers les enfants présentant un trachome actif et leur entourage proche, que vers les communautés. Ces résultats renforcent les initiatives de santé publique que constituent l'éducation sur la propreté du visage et les améliorations de l'environnement (stratégie CHANCE) pour combattre le trachome cécitant.
