# Supplementary material for: Active Trachoma among Children in Mali: Clustering and Environmental Risk Factors
Source: PLoS Negl Trop Dis. 2010 Jan 19;4(1):e583. doi: 10.1371/journal.pntd.0000583 (PMC2799671; doi:10.1371/journal.pntd.0000583)
Supplement: Table S1 — Risk factors for active trachoma (children under 10 years, Mali, 1996–1997), according to their hierarchical level and use. (0.08 MB DOC) [file pntd.0000583.s002.doc]

Table S1. Risk factors for active trachoma (children under 10 years, Mali, 1996-1997), according to their hierarchical level and use.

| **Variables** | **dismissed ^a^** | **not considered ^b^** | **considered ^c^** |
| --- | --- | --- | --- |
| Field-collected variables |  |  |  |
| Child level |  |  |  |
| Age |  |  | X |
| Dirty face |  |  | X |
| Flies on the face |  |  | X |
| Sex |  |  | X |
| Caretaker level |  |  |  |
| Caretaker |  |  |  |
| Education level |  |  | X |
| Nuptial status |  | X |  |
| Children |  |  |  |
| Frequency of body washing during the hot season | X |  |  |
| No. of children (all ages) |  | X |  |
| No. of children over 10 years |  | X |  |
| No. of children under 10 years |  | X |  |
| No. of face washings per day during the hot season |  |  | X |
| Use of soap for washing |  |  | X |
| Water quantity used per child | X |  |  |
| Wiping after washing |  |  | X |
| Water resources |  |  |  |
| Distance to the primary source of water |  | X |  |
| No. of months per year without water | X |  |  |
| Type of primary source of water |  |  | X |
| Type of secondary source of water | X |  |  |
| Household level |  |  |  |
| Household head |  |  |  |
| Education level |  |  | X |
| Exile history |  |  | X |
| Inhabitants |  |  |  |
| No. of caretakers |  |  | X |
| No. of children (all ages) |  |  | X |
| No. of children over 10 years |  |  | X |
| No. of children per family (all ages) | X |  |  |
| No. of children under 10 years |  | X |  |
| No. of children under 10 years per family | X |  |  |
| No. of families | X |  |  |
| No. of inhabitants | X |  |  |
| No. of people per bedroom | X |  |  |
| No. of people per family | X |  |  |
| Equipment |  |  |  |
| Bike |  |  | X |
| Car |  |  | X |
| Cart |  |  | X |
| Cows |  | X |  |
| Cowshed |  | X |  |
| Donkey |  | X |  |
| Garbage |  | X |  |
| House type | X |  |  |
| Latrines |  |  | X |
| Material wealth |  |  | X |
| Motorbike |  |  | X |
| No. of bedrooms | X |  |  |
| Plough |  |  | X |
| Radio |  |  | X |
| Roofing material |  |  | X |
| Sheepfold |  |  | X |
| Television |  |  | X |
| Traction bull |  |  | X |
| Well |  |  | X |
| Number of months with water | X |  |  |
| Use of latrines | X |  |  |
| Village level |  |  |  |
| Agriculture |  |  |  |
| Cotton production | X |  |  |
| Market gardening |  | X |  |
| Millet production | X |  |  |
| Peanut production | X |  |  |
| Rice production | X |  |  |
| Sorghum production | X |  |  |
| Tobacco production | X |  |  |
| Climate and vegetation |  |  |  |
| Aridity index |  | X |  |
| Budiko climate class |  |  | X |
| Gorczynski continentality index |  | X |  |
| Koeppen climate class |  | X |  |
| *Average annual conditions* |  |  |  |
| Daily temperature (maximum) |  | X |  |
| Daily temperature (mean) |  |  | X |
| Daily temperature (minimum) |  |  | X |
| Normalized difference vegetation index (maximum) |  | X |  |
| Normalized difference vegetation index (mean) |  |  | X |
| No. of months without rain |  | X |  |
| No. of rainy days |  |  | X |
| Precipitations |  |  | X |
| Sunshine fraction |  | X |  |
| Water vapor pressure |  |  | X |
| Wind speed |  |  | X |
| Average monthly conditions |  |  |  |
| Daily temperature (maximum) |  |  | X |
| Daily temperature (mean) |  |  | X |
| Daily temperature (minimum) |  | X |  |
| Normalized difference vegetation index (mean) |  |  | X |
| Number of rainy days |  |  | X |
| Precipitations |  | X |  |
| Sunshine fraction |  |  | X |
| Water vapor pressure |  |  | X |
| Wind speed |  | X |  |
| Equipment |  |  |  |
| No. of lined wells | X |  |  |
| No. of tube wells |  | X |  |
| No. of unlined wells | X |  |  |
| Pharmacy |  |  | X |
| School |  |  | X |
| Geography |  |  |  |
| Altitude |  |  | X |
| Distance to administrative center |  | X |  |
| Distance to health post | X |  |  |
| Distance to medical center |  | X |  |
| Latitude |  |  | X |
| Longitude |  |  | X |
| No. of backwaters |  | X |  |
| Permanent waterway in the vicinity |  |  | X |
| Socio-demography |  |  |  |
| No. of households | X |  |  |
| No. of inhabitants |  | X |  |
| Main ethnic group |  | X |  |
| Village association |  | X |  |
| Weekly market | X |  |  |
| Women’s association |  |  | X |

^a^ missing data exceeding a proportion of 10%

^b^ not considered in multivariate analyses (*p* value > 0.20 in univariate analyses)

^c^ considered in multivariate analyses (*p* value ≤ 0.20 in univariate analyses)
